# Exploring Dance as a Therapeutic Approach for Parkinson Disease Through the Social Robotics for Active and Healthy Ageing (SI-Robotics): Results From a Technical Feasibility Study

**DOI:** 10.2196/62930

**Published:** 2025-01-14

**Authors:** Roberta Bevilacqua, Elvira Maranesi, Marco Benadduci, Gabriella Cortellessa, Alessandro Umbrico, Francesca Fracasso, Giovanni Melone, Arianna Margaritini, Angela La Forgia, Pierpaolo Di Bitonto, Ada Potenza, Laura Fiorini, Carlo La Viola, Filippo Cavallo, Alessandro Leone, Andrea Caroppo, Gabriele Rescio, Mauro Marzorati, Amedeo Cesta, Giuseppe Pelliccioni, Giovanni Renato Riccardi, Lorena Rossi

**Affiliations:** 1 Scientific Direction, IRCCS INRCA, Via Santa Margherita 5, Ancona, 60124, Italy, 39 0718004767; 2 National Research Council–Institute of Sciences and Technologies for Cognition, Rome, Italy; 3 Exprivia S.p.a., Innovation Lab, Innovation, Marketing & Technology, Molfetta, Italy; 4 Department of Soil, Plant and Food Science, University of Bari Aldo Moro, Bari, Italy; 5 Grifo multimedia Srl, Bari, Italy; 6 Department of Industrial Engineering, University of Florence, Florence, Italy; 7 The BioRobotics Institute, Scuola Superiore Sant’Anna, Pontedera, Italy; 8 National Research Council of Italy – Institute for Microelectronics and Microsystems, Lecce, Italy; 9 Institute of Biomedical Technologies, National Research Council, Segrate, Italy; 10 Clinical Unit of Neurology, Istituto di Ricovero e Cura a Carattere Scientifico Istituto Nazionale Ricovero e Cura per Anziani (IRCCS INRCA), Ancona, Italy; 11 Clinical Unit of Physical Rehabilitation, Istituto di Ricovero e Cura a Carattere Scientifico Istituto Nazionale Ricovero e Cura per Anziani (IRCCS INRCA), Ancona, Italy

**Keywords:** Parkinson disease, rehabilitation, Irish dancing, balance, gait, socially interacting robot

## Abstract

**Background:**

Parkinson disease (PD) is a progressive neurodegenerative disorder characterized by motor symptoms. Recently, dance has started to be considered an effective intervention for people with PD. Several findings in the literature emphasize the necessity for deeper exploration into the synergistic impacts of dance therapy and exergaming for PD management. Moreover, socially engaging robotic platforms equipped with advanced interaction and perception features offer potential for monitoring patients’ posture and enhancing workout routines with tailored cues.

**Objective:**

This paper presents the results of the Social Robotics for Active and Healthy Ageing (SI-Robotics) project, aimed at designing an innovative rehabilitation program targeted at seniors affected by (early-stage) PD. This study therefore aims to assess the usefulness of a dance-based rehabilitation program enriched by artificial intelligence–based exergames and contextual robotic assistance in improving motor function, balance, gait, and quality of life in patients with PD. The acceptability of the system is also investigated.

**Methods:**

The study is designed as a technical feasibility pilot to test the SI-Robotics system. For this study, 20 patients with PD were recruited. A total of 16 Irish dance–based rehabilitation sessions of 50 minutes were conducted (2 sessions per week, for 8 wks), involving 2 patients at a time. The designed rehabilitation session involves three main actors: (1) a therapist, (2) a patient, and (3) a socially interacting robot. To stimulate engagement, sessions were organized in the shape of exergames where an avatar shows patients the movements they should perform to correctly carry out a dance-based rehabilitation exercise.

**Results:**

Statistical analysis reveals a significant difference on the Performance-Oriented Mobility Assessment scale, both on balance and gait aspects, together with improvements in Short Physical Performance Battery, Unified Parkinson Disease Rating Scale–III, and Timed Up and Go test, underlying the usefulness of the rehabilitation intervention on the motor symptoms of PD. The analysis of the Unified Theory of Acceptance and Use of Technology subscales provided valuable insights into users’ perceptions and interactions with the system.

**Conclusions:**

This research underscores the promise of merging dance therapy with interactive exergaming on a robotic platform as an innovative strategy to enhance motor function, balance, gait, and overall quality of life for patients grappling with PD.

## Introduction

Parkinson disease (PD) is a progressive neurodegenerative disorder characterized by motor symptoms, such as tremors, bradykinesia, rigidity, and postural instability, as well as nonmotor symptoms, including cognitive impairment, mood disorders, and autonomic dysfunction [1]. These symptoms can significantly impact patients’ quality of life and independence as the disease progresses. While pharmacological interventions, such as dopamine replacement therapy, remain the mainstay of PD management, their efficacy may diminish over time and they often fail to address the nonmotor symptoms adequately [2]. Physical therapy represents the key adjuvant treatment for PD, offering potential benefits in improving motor function, balance, gait, and overall mobility [3]. Among various forms of physical therapy, dance-based interventions have gained attention due to their multifaceted approach targeting motor, cognitive, and psychosocial aspects of the disease [4]. Dance therapy engages patients in rhythmic movements, music, and social interaction, promoting coordination, flexibility, and emotional well-being [5].

Exergaming, which involves interactive video games requiring physical movement, has emerged as a novel approach to delivering dance-based therapy in both home-based and clinical settings [6]. Exergames offer the advantages of personalized, adaptable, and enjoyable exercise programs, potentially enhancing patient motivation and adherence [7]. Recent research has highlighted the potential of dance therapy and exergaming to address the complex needs of PD patients. A randomized controlled trial by Duncan and Earheart [4] demonstrated significant improvements in motor function and balance among patients with PD participating in community-based dancing compared with a control group. Furthermore, a study by Barry et al [7] found that exergaming interventions were effective in improving mobility and reducing fall risk in patients with PD.

These findings underscore the importance of further investigating the combined effects of dance therapy and exergaming in PD management. Furthermore, the reliability and performance of current sensing technologies provide physiological data that are useful to monitor the state and the quality of the exercises objectively performed by patients. For example, commercial wearable devices could be easily integrated during rehabilitation sessions to gather information about the heart or breath rate of involved patients and objectively evaluate the metabolic effort. Socially interactive robotic platforms with their interaction and perception capabilities (eg, onboard cameras) are well suited to monitor the posture of patients and support the execution of the exercises through tailored stimuli. A more comprehensive approach may integrate advanced technologies, such as sensor technology for real-time monitoring, artificial intelligence (AI) techniques for personalized difficulty adjustments, and robotic platforms to deliver personalized feedback and enhance the rehabilitation experience.

In this context, the Social Robotics for Active and Healthy Ageing (SI-Robotics) project designed an innovative rehabilitation program targeted at seniors affected by (early stage) PD [8]. The approach relies on the integration of several AI technologies ranging from knowledge representation and reasoning, for user modeling and personalization to machine learning and automated planning (AP) for continuous proactive and adaptive assistance [9]. Leveraging the mentioned interventions based on different types of dance [10,11], the idea is to develop a technology-enhanced, dance-based rehabilitation program where a socially interacting robot and several sensing devices (wearable sensors and 3D cameras mainly) support therapists and patients during the execution of the exercises. This study therefore aims to assess the usefulness of a dance-based physical therapy program enriched by AI-based exergames and contextual robotic assistance in improving motor function, balance, gait, and quality of life in patients with PD. We hypothesized that the proposed technology-enhanced dance therapy would lead to greater improvements in PD symptoms.

## Methods

The study is designed as a technical feasibility pilot to test the SI-Robotics system. The entire protocol, including the description of scales, the platform functioning, the training program, and procedures has been previously described in detail [8].

### Subjects

A total of 20 patients with PD were selected by the outpatient department at the Clinical Unit of Physical Rehabilitation, Istituto di Ricovero e Cura a Carattere Scientifico Istituto Nazionale Ricovero e Cura per Anziani (IRCCS INRCA). The patients were recruited if they were over 65 years old; able to provide informed consent; had a stage of Hoen and Yahr scale between 1 and 2 [12]; had a Functional Ambulation Category score ≥2 [13]; had a Rankin Scale score ≤3 [14]; had stability of drug treatment for at least 1 month; had a Geriatric Depression Scale 4-item score ≤1 [15]; had a Mini-Mental State Examination ≥24 [16]; and could maintain an upright posture≥30 seconds, evaluated by a trained physiotherapist during the recruitment. The evaluation of compliance with the inclusion and exclusion criteria was performed during the recruitment session. Once we completed this phase, informed consent was obtained. The patient’s clinical assessment was performed at the start and the end of the treatment. In particular, the evaluation consisted of the administration of the following scale: measurement of functional state with the Barthel Index [17]; physical performance with Tinetti’s Performance-Oriented Mobility Assessment (POMA) [18], the Short Physical Performance Battery (SPPB) [19], the 6-Minute Walking Test [20], and the Timed Up and Go test (TUG) [21]; evaluation of the quality of life with the 12-Item Short-Form Health Survey (SF-12) [22]; fear of falling with Falls Efficacy Scale-International (FES-I) [23]; and the assessment of the prognosis of PD with the Unified Parkinson Disease Rating Scale – III (UPDRS-III) [24].

### Intervention

**Figure 1.**
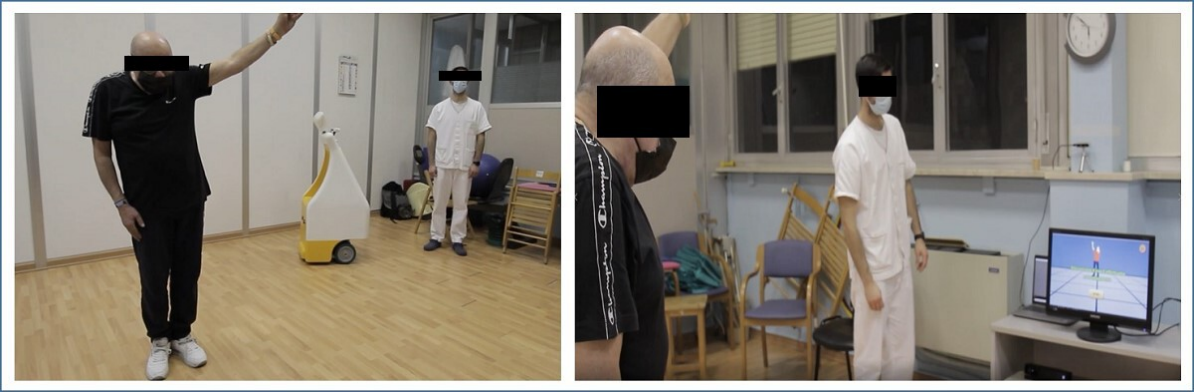
Technological components and actors of Social Robotics for Active and Healthy Ageing (SI-Robotics) rehabilitation sessions.

Figure 1 shows the experimental setting in a protected environment (gymnasium) and the positioning of the technologies, the patient, and the physiotherapist, during the dance-based rehabilitation sessions.

A total of 16 therapy sessions of 50 minutes were conducted (2 sessions per wk, for 8 wks), involving 2 patients at a time. Cardiac and respiratory activity was monitored during dancing treatments to detect heart rate and breathing frequency. The participants were required to complete at least 80% of the sessions. Each session involved the following activities: (1) breathing, relaxation, and postural harmonization exercises (5 min); (2) active mobility and stretching exercises (5 min); (3) the SI-Robotics intervention (35 min) consisting of the AI-enhanced “Let’s Dance” game, and a socially interacting robot monitoring the execution of the physical exercises of patients; and (4) relaxation exercises (5 min).

After selecting a difficulty level, players are presented as dancers on the screen, along with footprints that suggest the movement to be done. Each task can vary from simple aerobic exercises (side steps, arm raising, hand clapping, etc) to choreographies. At the end of the game session, a score is presented to the patient. This would make a patient aware of the quality of the performance. This score can also be used by the therapist to assess the patient’s performance and the possible increase in the difficulty level of the game.

The next sections describe the technological components of the session in more detail.

### The AI-Based Rehabilitation Session

The designed rehabilitation session involves three main actors: (1) a therapist, (2) a patient, and (3) a socially interacting robot. To stimulate engagement, sessions are organized in the shape of exergames where an avatar shows patients the movements they should perform to correctly carry out a rehabilitation exercise (eg, dancing steps). Given a set of data about the needs of a group of patients and about stimulation capabilities of known exercise, a game engine integrates an AI planner [25] and synthesizes exercises by selecting a (sub)set of movements (ie, dancing steps) that best fit the clinical objectives of the session (ie, of the current patient). The exergame is enriched with AP to synthesize suitable physical exercises, contextualized to the clinical objectives of the rehabilitation sessions [25]. Many works in the literature investigate the use of AI in health care and PD [11,26] AI is used for example to predict the wearing-off of symptoms [27], support decisions [28], or early diagnosis of PD [29]. The majority of these works adopt AI solutions based on deep or machine learning and focus on the diagnosis of the disease. Few works investigate the use of AP to support therapists in the synthesis of rehabilitation programs. The study by Gonzalez et al [30], for example, integrates AP into a control architecture to allow a social robot to physically show motions to a patient during physical rehabilitation. Baschieri et al [31] uses AP to synthesize simulation scenarios within a serious game for cognitive rehabilitation. This work pursues an objective similar to ours but in a different clinical scenario.

As shown in Umbrico et al [25] SI-Robotics proposes AP to support the synthesis of physical rehabilitation programs for patients with PD . A key aspect of the developed planning framework is the combined reasoning about spatial and clinical effects of stimuli (ie, motions or dancing steps) needed to synthesize plans that are technically valid and effective from a clinical point of view. Compared with a manual definition of the rehabilitation session, the use of a planner aims to improve the quality, accuracy, and engagement of the resulting rehabilitation programs. A therapist provides a planner with data about the rehabilitation session (song time, song rhythm, and difficulty level) and the clinical objectives. Given this input, the planner decides a sequence of steps, optimized according to an objective function encoding the specified clinical objective. Planned steps are thus chosen according to the rehabilitation needs of the participating patient (personalization).

**Figure 2.**
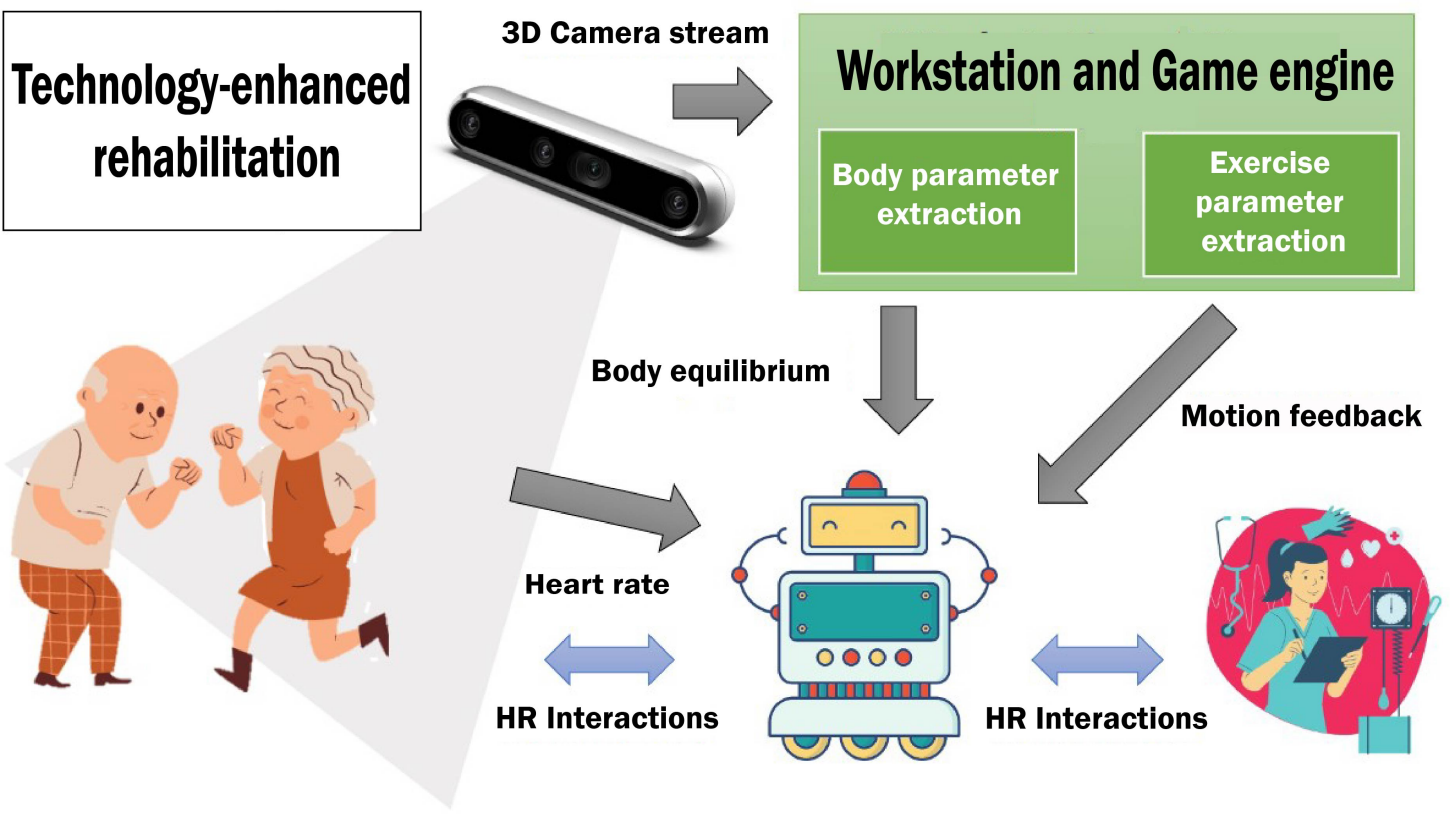
Automata describing the assistive behavior of the robot during a rehabilitation session. HR: Human-Robot

In addition, several devices enrich the session to extract useful data about the health conditions and the performance of patients. The generated data streams are gathered by the robot that embodies the AI-based reasoning modules. Overall, three types of perception components were considered: (1) wearable sensing devices (eg, sensorized shirts) that constantly produce data streams about physiological parameters of patients (eg, heart rate) [32]; (2) a video-processing component elaborating 3D camera data to extract kinematics about motions and produce data about body posture and body equilibrium of patients and; (3) a video-processing component elaborating 3D camera data to analyze patients’ behaviors and produce data about the correctness of performed motions (ie, feedback). Figure 2 below shows a conceptual overview of the designed session pointing out a subset of the main hardware and software components and the data streams generated during the execution of a rehabilitation session.

**Figure 3.**
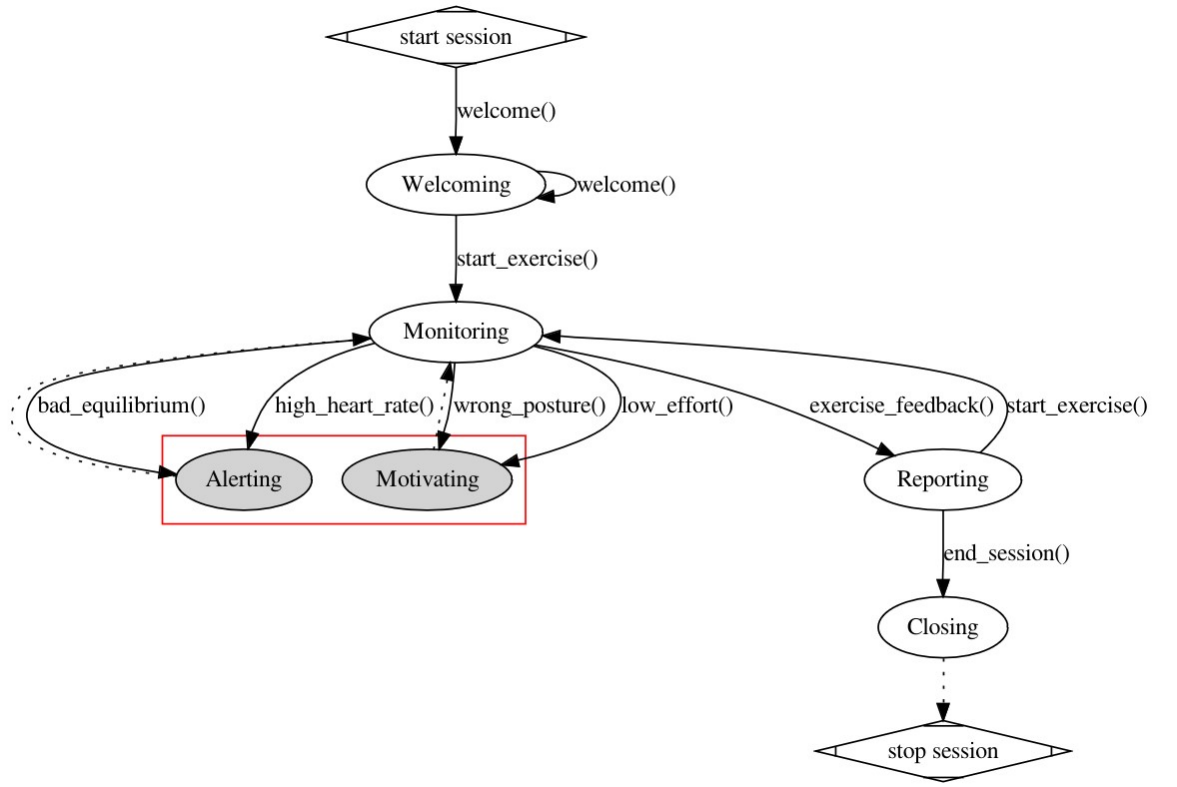
Architectural structure of the artificial intelligence–based cognitive control of the robot.

In addition, a socially interacting robot should support the execution of a session by recognizing the current state of a session according to data gathered from the environment, autonomously detecting events that would trigger different phases, and setting the behavioral goals needed to support a session. The robotic platform consists of a novel social robotic platform designed within SI-Robotics and developed by Co-Robotics [33]. Figure 3 shows the lifecycle determining the assistive behavior of the robot during each rehabilitation session.

A rehabilitation session starts with a welcoming state where the robot introduces patients to the session. Patients do not know the structure of the rehabilitation and are not familiar with the involved technology. The objective of this state is therefore to prepare patients for the therapy by explaining the structure of the session and the technology used. When all patients are ready to start a session, the robot enters into a “monitoring” state gathering data from the environment to monitor the performance and health state of patients. When a rehabilitation exercise ends the robot enters into the “reporting” state to interact with the therapist to comment on the exercise and the observed performance. This state is especially useful to enrich the subjective experience of the therapist by enhancing the awareness of the therapist with objective feedback on the quality of the exercises. It is indeed meant as a support for the decisions on how to proceed with the session (eg, change or repeat the exercises, difficulty of the next exercise, etc). If the therapist decides to start a new exercise, then the robot enters again into the monitoring state. Otherwise, the robot enters into the “closing” state to finalize the session. In this latter case, the robot interacts with patients to ask their personal feelings about the session and to show a qualitative assessment of their session.

Different types of interaction are foreseen during an exercise, depending on the type of situation recognized by the robot. Two types of interactions have been considered characterized by the motivating and the alerting states in the automata. The motivating phase allows the robot to play the role of a coach during the session stimulating patients to perform better or correcting their behavior. The robot is supposed to enter the motivating phase when it detects a heart rate below some minimum “training threshold” or when it detects a wrong posture of the body, for example, bad alignment or asymmetrical motion of lower or upper limbs.

The alerting phase allows the therapist (and the robot) to intervene when some critical situation concerning the health state of a patient is detected during the session. In such case, the robot is supposed to warn the therapist about the danger and simultaneously interact with the involved patient, even interrupting the exercise if necessary. Specifically, the robot enters the alerting phase when it detects the risk of a fall or an anomalous heart rate for a patient. Both these situations are critical for the considered target of patients and need a prompt intervention.

### AI-Based Control of Robotic Skills

**Figure 4.**
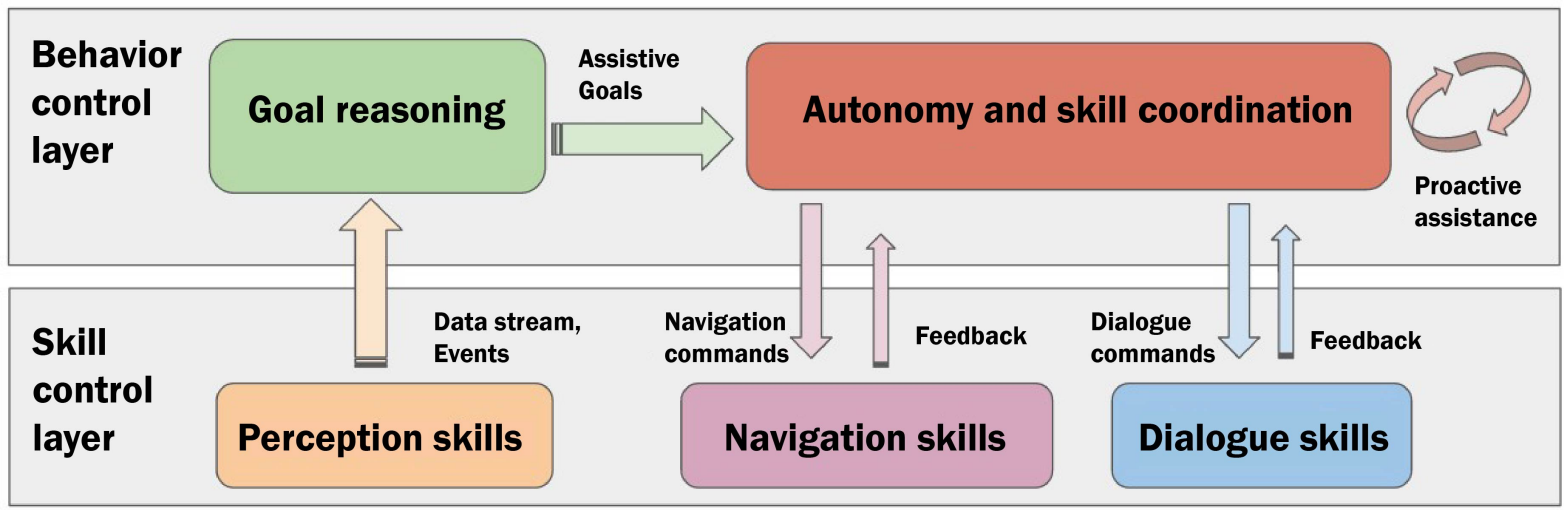
Designed architecture.

A key aspect (and still an open challenge) in the design of autonomous robotic agents is the integration of different AI technologies and Robotics [34,35]. SI-Robotics proposes an AI-based control architecture with twofold objectives: (1) to support the abstraction and reasoning capabilities needed to recognize health-related situations and assistive objectives and (2) to coordinate robotic skills to “act” in the environment and (autonomously) achieve contextualized assistive objectives. Figure 4 shows an overview of the designed architecture with the main functional components involved in the control process. Two main architectural layers are considered and are organized according to a cognitive architecture inspired by the Dual Process Psychological Theory [36].

A skill control layer encapsulates the control components responsible for providing the robot with the “low-level” capabilities needed to perceive the environment and interact with the environment. The Navigation Skills component encapsulates functionalities that allow a robot to robustly navigate within the environment and autonomously reach known locations. The Dialog Skills encapsulate natural language processing functionalities that allow a robot to talk with patients and therapists. This component in particular enables an affective interaction [37] by adapting the way the robot talks with a patient to the detected emotional state and known personality traits.

While some level of perception is needed for both navigation and dialogue skills, the Perception Skills component emphasizes the additional capability needed to process data from deployed environmental devices. It encapsulates low-level data processing to provide the higher decisional level with refined information. A behavior control layer supports AI-based control features to contextualize observations, trigger assistive goals, and coordinate underlying skills to implement the desired assistance. The Autonomy and Skill Coordination component encapsulates an AI plan–based controller that supports behavior continuity coordinating robotic skills. The Goal Reasoning component instead is in charge of contextualizing observations to automatically set assistive goals. The combination of goal reasoning and a plan-based controller is the key architectural feature enabling proactive and autonomous goal-oriented behaviors of the robot [38].

The AI-based architectural elements composing the coordination layer of Figure 4 have been embedded into the robotic platform and integrated through the Robot Operating System (ROS) using the ROS Melodic distribution [39]. The coordination component responsible for the synthesis of the assistive behavior has been implemented in the shape of an AI plan–based controller relying on AP. The component uses the open-source framework ROXANNE [40], which implements goal-oriented acting capabilities into an ROS [41]. ROXANNE allows the robot to receive high-level assistive goals and (autonomously) synthesize and execute navigation and dialogue commands or tasks to realize a desired assistive behavior. The goal reasoning component correlates data received from the perception component with the autonomous behavior of the robot. It endows the robot with the cognitive capabilities needed to understand the evolution of a rehabilitation session autonomously select suitable planning goals and consequently decide the assistance needed in a particular context.

Embedded AP models the behavioral constraints needed to correctly support a rehabilitation session. Behavioral constraints are specified in the shape of temporal constraints and require interacting tasks performed through available skills. In this regard, Table 1 shows the modeled assistive goals and the parameters defined to contextualize interactions. Furthermore, the table briefly describes the behavioral constraints specified in the model to correctly implement the desired assistance and support the associated goal. It is worth noting that AI planning facilitates robot programming since it supports an “easy specification” of desired behavioral dynamics without hard-coding the implemented skills.

**Table 1.** Acting goals supported by the robot-embedded controller to synthesize assistive behaviors during a rehabilitation session.

Goal	Interaction parameters	Interaction parameters
Welcoming	Therapist: string Patient: string Gender: string Novel: boolean	The robot moves from its current location to the welcoming area to call the specified patient and take patient to the dressing area to wear the sensorized shirt. Then it guides the patient to the rehabilitation area to start the session. If the patient is new to the session (flag novel set to true) the robot explains the organization of the rehabilitation and the functioning of the devices. Otherwise, the robot shows data about the last session to motivate the patient for the current one.
Exercise start	—^a^	When an exercise begins the robot starts moving around the rehabilitation area and monitoring patients’ state by collecting physiological, body posture, and performance data.
Exercise report	Patient: string Gender: string Score: double Past_score: double Global_score: double	When an exercise ends the robot moves close to a patient to show a brief report about the patient performance. The robot shows the achieved score and (if available) compares it to the average score of the patient in the past and the average score of other patients. This would make a patient aware of the quality of the performance.
Low heart rate and high heart rate	Therapist: string Patient: string Gender: string Heart_rate: double	The robot periodically analyzes trends of gathered data about the heart rate of a patient. Thresholds are parametric and computed according to the age of the patient as follows: HR_max=80% (220 – age) HR_min=40% h_max The objective of the session is to keep the heart rate of patients within HR_max and HR_min to stimulate a proper metabolic effort. In the case of low heart rate (i.e., value below HR_min) the robot implements a motivational behavior asking a patient to improve the performance. In case of high heart rate (i.e., value above HR_max), the root implements an assistive behavior alerting the therapist and asking the patient to reduce the performance
Bad posture	Patient: string Gender: string Tilt: integer in [0, 1] Arms: integer in [–1, 1] Legs: integer in [–1, 1]	The robot constantly monitors the motions and the inclination of patients. If anomalous kinematics parameters are detected, for example, wrong inclinations of the body or wrong alignment of arms or legs, the robot implements a motivational behavior to correct the body posture of a patient and thus stimulate correct motions.
Bad equilibrium	Therapist: string Patient: string Gender: string Lateral: integer in [–1,1] Frontal: integer in [–1,1]	In addition to the body posture, the robot constantly monitors the equilibrium of patients to prevent dangerous falls during physical therapy. If dangerous equilibrium conditions are detected from camera data (eg, the center of mass is outside the patient’s base) the robot implements an assistive behavior to promptly notify the therapist and warn the patient about the danger or falling.

a Not applicable.

### Outcomes

All assessment procedures adhere to a standardized protocol. Specifically, the primary focus of the study revolves around enhancing balance, and gait, and alleviating the fear of falling among elderly patients with PD. This is gauged through the utilization of the 3 POMA scales (POMA balance, POMA Gait, and POMA Total) after the 10 treatment sessions, as a result of the Irish dance intervention. In addition, secondary measures include evaluating the gait speed of elderly patients with PD, their fear of falling (assessed via FES-I), their physical performance (SPPB), their autonomy in daily living activities (UPDRS-III), and their overall physical and psychological well-being (SF-12) together with the evaluation of the Unified Theory of Acceptance and Use of Technology (UTAUT) that is composed by 10 subscales (anxiety; attitude; facilitating conditions; intention to use; perceived adaptability; perceived enjoyment; perceived ease of use; perceived usefulness; social influence; and trust).

### Statistical Analysis

Continuous variables were presented as either mean and SD or median and IQR, depending on their distribution, which was determined using the Kolmogorov-Smirnov test. Categorical variables were expressed as absolute numbers and percentages. To test statistically significant differences (*P*<.050) between pre- and postconditions, Wilcoxon signed-rank test (for non-normal distribution) or paired test (for normal distribution) were used, alongside simple descriptive statistics such as means, medians, and SDs as appropriate. The statistical analysis was performed using SPSS software.

### Ethical Considerations

The study was approved by the Ethics Committee of the Istituto Nazionale Ricovero e Cura per Anziani, (IRCCS INRCA) on June 17, 2021 (CE INRCA 21004). The protocol is registered on ClinicalTrials.gov with trial registration number NCT05005208 (October 23 , 2023). All participants signed the informed consent and data processing consent. The data are anonymised so that the identity of the subject cannot be traced. No compensation is provided for participation in the study.

## Results

Demographic and clinical data of the patients are reported in Table 2. Two participants dropped out because they did not complete the rehabilitation program.

**Figure 5.**
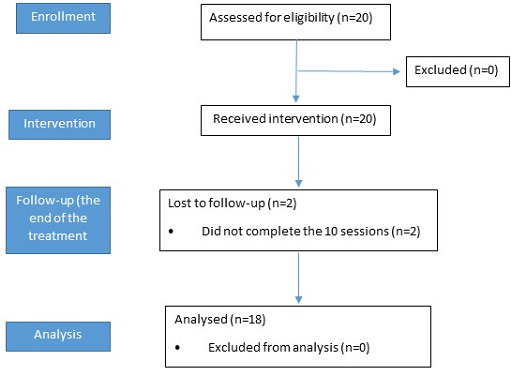
The CONSORT (Consolidated Standards of Reporting Trials) flowchart.

The CONSORT (Consolidated Standards of Reporting Trials) flowchart is shown in Figure 5.

Gender differences were not statistically significantly different in all scales used to select the sample.

**Table 2.** Baseline demographic and clinical profile.

		Total (n=18)	Male (n=10)	Female (n=8)	*P* value
Age (years), mean (SD)		75.3 (5.5)	73.7 (5.4)	77.2 (5.2)	.18
**Marital status, n (%)**					.48
	Married	15 (83.3)	9 (90)	6 (75)	
	Single	1 (5.5)	0 (0)	1 (12.5)	
	Widowed	2 (11.2)	1 (10)	1 (12.5)	
**Educational level, n (%)**					.17
	Primary education	4 (22.2)	1 (10)	3 (37.5)	
	Secondary education	10 (55.6)	6 (60)	4 (50)	
	University or more	4 (22.2)	3 (30)	1 (12.5)	
Hoehn and Yahr score, mean (SD)		1.8 (0.3)	1.9 (0.3)	1.7 (0.4)	.42
Rankin Scale score, mean (SD)		1.6 (0.9)	1.4 (0.8)	1.8 (1.1)	.33
GDS^a^, mean (SD)		3.0 (0.7)	2.9 (0.7)	3.2 (0.7)	.33
FAC^b^, mean (SD)		4.6 (0.6)	4.7 (0.4)	4.5 (0.74)	.50
MMSE^c^, mean (SD)		29.4 (0.9)	29.7 (0.6)	29.1 (0.7)	.22
Barthel Index score, mean (SD)		95.5 (5.6)	94.5 (6.8)	96.8 (3.7)	.39

a GDS: Geriatric Depression Scale.

b FAC: Functional Ambulation Category.

c MMSE: Mini-Mental State Examination.

Table 3 shows differences between pre- and postintervention on the functional state scales with the UPDRS-III and the SPPB, gait and balance performance on Tinetti POMA Gait and POMA Balance, evaluation of quality of life with SF-12 and its subscores (physical component score and mental component score), fear of falling (FES-I) together with the TUG execution time, the meters covered during the 6-Minute Walking Test and the gait speed.

Statistical analysis revealed a significant effect on UPDRS-III and TUG together with improvements in the POMA scale, both on balance and gait aspects, and in SPPB. However, for the POMA and SPPB scales, there was a low Cohen *d* coefficient, underlining a probable effect of the low sample size on these results. In contrast, the effect size for UPDRS-III and TUG was high (>0.70), highlighting the usefulness of the rehabilitation intervention on the motor symptoms of PD also in our small sample.

**Table 3.** Mean (SD) values of the mean of pre- and postintervention scores on the UPDRS-III^a^, SPPB^b^, POMA^c^ (total, gait and balance), SF-12^d^ (total, physical, and mental component score), FES-I^e^, TUG^f^ execution time, 6MWT^g^, and gait speed. Pre-post and between-group comparisons are reported for each score (*P*<.05). The effect size (Cohen *d*) is also reported.

		Preintervention scores, mean (SD)	Postintervention scores, mean (SD)	*P* value	Cohen *d*
UPDRS-III		13.56 (1.85)	13.83 (1.85)	.01 ^h^	−0.73
SPPB		8.83 (2.61)	10.39 (2.45)	.02 ^h^	0.10
**POMA**					
	POMA Total	24.11 (3.92)	26.28 (2.74)	.001^h^	−0.07
	POMA Gait	9.83 (2.30)	10.94 (1.55)	.01 ^h^	−0.02
	POMA Balance	14.28 (2.16)	15.33 (1.37)	.01 ^h^	−0.10
**SF-12**					
	SF-12-Tot^i^	31.28 (1.99)	32.33 (2.42)	.10	−0.20
	PCS-12^j^	13.56 (1.84)	13.83 (1.85)	.49	−0.23
	MCS-12^k^	17.72 (1.22)	18.50 (1.91)	.12	−0.16
FES-I		10.78 (1.92)	10.67 (2.22)	.83	−0.29
TUG (s)		11.0 (3.91)	9.19 (3.14)	.002^h^	−0.82
6MWT (m)		361.50 (99.33)	373.72 (98.67)	.40	−0.19

a UPDRS-III: Unified Parkinson Disease Rating Scale – III.

b SPPB: Short Physical Performance Battery.

c POMA: Performance Oriented Mobility Assessment.

d SF-12: 12-Item Short-Form Health Survey.

e FES-I: Falls Efficacy Scale–International.

f TUG: Timed Up and Go test.

g 6MWT: 6-Minute Walking test.

h *P* values from matched-pairs Student *t* test.

i SF-12-Tot: 12-Item Short-Form Health Survey total score.

j PCS-12: 12-Item Short-Form Health Survey physical component score

k MCS-12: 12-Item Short-Form Health Survey mental component score.

Table 4 reports the scores of the subscales of UTAUT.

The analysis of the UTAUT subscales provided valuable insights into users’ perceptions and interactions with the system. In particular, attitude, perceived adaptability, enjoyment, ease of use, usefulness, and social influence were generally positive.

**Table 4.** Mean (SD) of the mean of the Unified Theory of Acceptance and Use of Technology (UTAUT) subscales scores.

UTAUT subscales		Score, mean (SD)	Range
Anxiety (ANX)	Evoking anxious or emotional reactions when using the system.	7.4 (4.2)	0‐20
Attitude (ATT)	Positive or negative feelings about the appliance of the technology	11.2 (2.5)	0‐15
Facilitating conditions (FC)	Objective factors in the environment that facilitate using the system	2.6 (1.7)	0‐15
Intention to use (ITU)	The outspoken intention to use the system over a longer period in time	3 (0)	0‐10
Perceived adaptability (PAD)	The perceived ability of the system to be adaptive to the changing needs of the user.	10.5 (3.0)	0‐15
Perceived enjoyment (PENJ)	Feelings of joy or pleasure associated by the user with the use of the system.	18.2 (2.2)	0‐25
Perceived ease of use (PEOU)	The degree to which the user believes that using the system would be free of effort	18.1 (3.8)	0‐25
Perceived usefulness (PU)	The perceived ability of the system to perform sociable behavior.	11.4 (2.9)	0‐15
Social influence (SI)	The user’s perception of how people who are important to him think about him using the system	8 (2.4)	0‐10
Trust	The belief that the system performs with personal integrity and reliability.	7.5 (1.8)	0‐10

## Discussion

### Principal Findings

This study aims to investigate the usefulness of the SI-Robotics intervention based on AI robotic assistance and exergame stimulation, in the context of PD. This study aims to assess the ability of a dance-based physical therapy program enriched by AI-based exergames and contextual robotic assistance in improving motor function, balance, gait, and quality of life in patients with PD. The findings of this study demonstrate the potential usefulness of a dance-based physical therapy program using a robotic platform to deliver exergames in improving motor function, balance, gait, and quality of life in patients with PD. Results suggest that integrating dance therapy with engaging technology may lead to greater improvements in PD symptoms compared with conventional physical therapy alone. Although the statistical analysis revealed significant improvements in motor function, balance, and gait (eg, UPDRS-III, POMA, and TUG scores), the effect sizes for several outcome measures were relatively small (Cohen *d*<0.2 for POMA and SPPB scales). This suggests that, while the observed changes were statistically significant, the practical impact of the intervention on certain motor and functional outcomes may be limited. These small effect sizes could be partly attributed to the small sample size, which may have reduced the power to detect larger effects.

This study adds to the growing body of research supporting the use of dance therapy and exergaming as adjunctive treatments for PD. Previous studies have shown that both dance therapy and exergaming can independently improve motor function, balance, and mobility in patients with PD [4,7]. By combining these approaches, our intervention aimed to target the multifaceted nature of PD symptoms, including motor, cognitive, and psychosocial aspects.

The results are consistent with previous findings demonstrating the benefits of dance therapy and exergaming in PD management. For example, Duncan and Earheart [4] reported significant improvements in motor function and balance among patients with PD participating in community-based dancing, while Barry et al [7] found that exergaming interventions were effective in improving mobility and reducing fall risk in patients with PD. These studies, along with ours, highlight the potential of integrating dance therapy with exergaming to address the complex needs of patients with PD.

One novel aspect of our intervention is the use of the SI-Robotics system, a robotics-based platform designed to engage patients with PD in Irish set dancing. This innovative approach combines the physical and cognitive benefits of dance therapy with the interactive and adaptable nature of exergaming. By incorporating personalized avatars and choreography, the “Let’s Dance” game provided a stimulating and enjoyable exercise experience for participants, potentially enhancing motivation and adherence to the intervention.

Regarding the analysis of technology acceptance among the population studied, the results indicate a generally positive perception and interaction with technology. In particular, users displayed a positive attitude towards the technology, indicating favorable feelings toward it. They also found the system adaptable, enjoyable, easy to use and useful showing that users experienced pleasure while using the system. Finally, trust in the system was moderately high, indicating that users believed it to be reliable and trustworthy. These highlight how the system successfully meets user expectations by offering an engaging, straightforward, and beneficial experience.

Our study focused on elderly patients with PD with mild to moderate disease severity, as reflected by their scores on the Hoen and Yahr scale, Functional Ambulation Category, Rankin Scale, Geriatric Depression Scale, and Mini-Mental State Examination. These inclusion criteria aimed to ensure that participants were physically and cognitively capable of engaging in the intervention safely and effectively. Future research could explore the applicability of our intervention to a broader range of patients with PD , including those with more advanced disease stages.

Limitations of our study include the small sample size and lack of a control group for comparison. While our results provide preliminary evidence of the usefulness of the intervention, larger-scale randomized controlled trials are needed to confirm these findings and establish the optimal dosage and timing of dance-based physical therapy with exergames for PD management. In addition, longer-term follow-up assessments could help evaluate the sustainability of the intervention effects over time.

### Conclusions

In conclusion, this study highlights the potential of integrating dance therapy with exergaming integrated with an interactive robotic platform as a novel approach to improving motor function, balance, gait, and quality of life in patients with PD. The SI-Robotics intervention offers a promising avenue for future research and clinical practice in the management of PD symptoms. To strengthen the validity of the current findings and ensure their broader applicability, future research will focus on replicating the study with a larger sample. This will help to verify the generalizability of the results providing a more comprehensive understanding of the observed effects. Moreover, further studies are warranted to explore the long-term effects and feasibility of implementing this intervention in diverse clinical settings.

## Abbreviations

AI: artificial intelligence
BI: Barthel Index
FES-I: Falls Efficacy Scale-International
PD: Parkinson disease
POMA: Tinetti’s Performance-Oriented Mobility Assessment
ROS: Robot Operating System
SF-12: 12-Item Short-Form Health Survey
SI-Robotics: Social Robotics for Active and Healthy Ageing
SPPB: Short Physical Performance Battery
TUG: Timed Up and Go
UPDRS-III: Unified Parkinson Disease Rating Scale – III
